# Regulatory T Cells Suppress Antiviral Immune Responses and Increase Viral Loads during Acute Infection with a Lymphotropic Retrovirus

**DOI:** 10.1371/journal.ppat.1000406

**Published:** 2009-08-28

**Authors:** Gennadiy Zelinskyy, Kirsten Dietze, Tim Sparwasser, Ulf Dittmer

**Affiliations:** 1 Institut für Virologie der Universitaet Duisburg-Essen, Essen, Germany; 2 Institute for Infection Immunology, TWINCORE, Centre for Experimental and Clinical Infection Research; a joint venture between the Medical School Hannover and the Helmholtz Centre for Infection Research, Hannover, Germany; The Scripps Research Institute, United States of America

In a recent paper in *Science*, Lund et al. [1] investigated the effect of regulatory T cells (Tregs) on the antiviral immune response to an acute herpes virus infection. Previous studies on the role of Tregs in different viral infections suggested that this T cell population suppresses antiviral effector T cell responses or local immune activation at the sites of viral replication [2],[3], which might subsequently facilitate viral immune evasion and the establishment of chronic infections [4],[5],[6]. Thus, it was quite surprising that ablation of Tregs in HSV-2-infected mice resulted in an accelerated fatal infection with increased viral loads instead of enhanced immunity to the virus [1]. In their fascinating paper, the authors showed that depletion of Tregs amplified the immune responses in draining lymph nodes at the site of infection, but at the same time delayed the entry of the immune cells into the HSV-2-infected tissue. As a general concept, Lund et al. postulated that Tregs promote immune responses in acute infections in which the pathogen replicates in non-lymphoid tissues. However, a number of viruses that induce severe human diseases, like HIV or the measles virus, replicate in primary or secondary lymphoid organs. Therefore, the question arises whether Tregs have a suppressive or promoting effect on antiviral immune responses in infections with viruses that are lymphotropic.

We have used the Friend retrovirus (FV) mouse model to address this question. FV is a lymphotropic retroviral complex that replicates efficiently in the spleen of infected mice and can induce a lethal erythroleukemia [7]. It was demonstrated in previous studies that the virus induces a significant expansion of Tregs during acute infection [8]. Similar to Lund et al. [1], we used a transgenic mouse expressing the diphtheria toxin (DT) receptor and GFP under the control of the Foxp3 promoter [9] to selectively deplete Tregs by DT injection during acute FV infection. DT injection into FV-infected mice at days 0, 2, 4, 6, and 8 postinfection depleted all GFP-expressing CD4^+^ Foxp3^+^ T cells in the spleen (Figure S1A), which resulted in a slight reduction of the overall CD4^+^ T cell counts (Figure S1B). However, a small population of cells positive for CD4 and Foxp3 but negative for GFP remained detectable. The DT injection did not significantly influence the overall numbers of CD8^+^ T cells and CD19^+^ B cells in the spleen of infected mice, indicating that the general lymphocyte population was not affected. In addition, a recent study clearly indicates that a DT injection into mice expressing a DT receptor/GFP cassette under the control of the Foxp3 promoter leads to a specific ablation of Tregs [10], but not to the depletion of epithelial cells as previously suggested by Liu et al. [11].

Since virus-specific CD8^+^ T cells are the most important immune cells that control acute FV replication [12], we determined the quantity and quality of these cells in the spleen of mice experimentally ablated of Tregs. At 10 days postinfection, significantly more CD8^+^ T cells expressing markers of effector T cells (CD43^+^) were found in the spleen of Treg-depleted mice compared to non-depleted controls (Figure 1A). Treg ablation also enhanced the number of FV-specific CD8^+^ T cells in the spleen of infected mice, which were stained with a FVgag MHC class I tetramer [4] (Figure 1B). In addition to the magnitude of the CD8^+^ T cell response, we analyzed the functional properties of these T cells. After Treg ablation, the expression of the cytotoxic molecules granzyme A and B in splenic CD8^+^ T cells was significantly enhanced during acute FV infection (Figure 1C and 1D), suggesting an improved cytotoxic potential of these cells. To verify that the enhanced granzyme production was associated with an improved degranulation of cytotoxic molecules by CD8^+^ T cells, expression of the degranulation marker CD107a [13],[14] was also determined. Figure 1E shows that Treg cell depletion significantly increased the number of effector CD8^+^ T cells expressing CD107a. In addition, Treg ablation not only influenced the production of cytotoxic molecules by antiviral CD8^+^ T cells, but also influenced their cytokine response. After FV infection, subpopulations of the CD8^+^ T cells produced IFNγ, TNFα (Figure S2 and [15]), or low amounts of IL-2. Treg depletion increased the percentage of CD8^+^ T cells producing these cytokines after FV infection (Figure S2). In depleted mice most of the responding CD8^+^ T cells were multifunctional. Multifunctional T cells simultaneously produce two or more cytokines and have enhanced antiviral effector functions [16]. Most cytokine-producing CD8^+^ T cells from DT-treated DEREG mice expressed two or three different cytokines, whereas in non-depleted mice the majority of the effector CD8^+^ T cells expressed only one of the three cytokines measured (unpublished data). In contrast to the results from Lund et al. [1], the augmented virus-specific CD8^+^ T cell response decreased viral loads in the spleen more than ten times (Figure 1F). Thus, targeted depletion of Treg during acute FV infection resulted in superior control of viral replication rather than an accelerated infection as reported for HSV-2 [1]. The results clearly show that the suppressive activity of Tregs on antiviral immunity is the predominant effect in viral infections in which the primary targets of the virus are cells of the lymphoid organs. This suggests that the concept derived from the study of Lund et al. [1] might mainly apply to pathogens replicating in non-lymphoid tissue during acute infection. Therefore, inhibiting Treg responses therapeutically in infections with lymphotropic viruses might still be an interesting approach for antiviral treatment.

**Figure 1:**
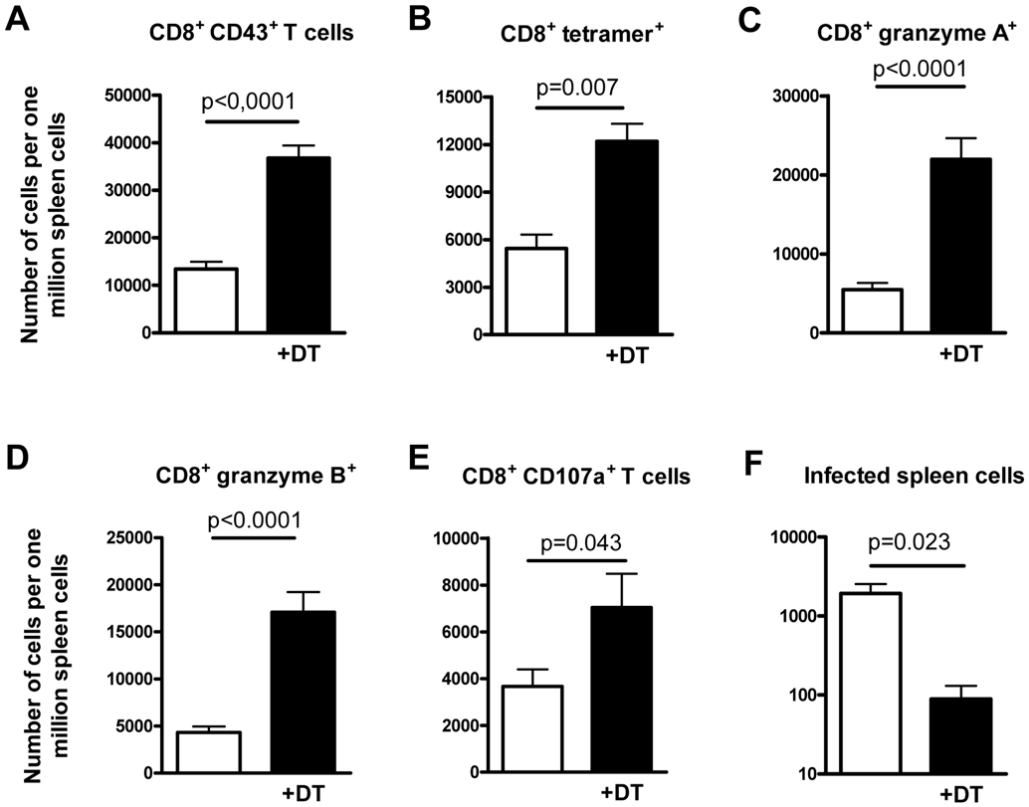
Cytotoxic CD8^+^ T cell responses and spleen viral loads in mice infected with FV and experimentally depleted of Tregs. DEREG mice [9] were infected with FV and Tregs were depleted starting at the time point of infection by five injections (days 0, 2, 4, 6, and 8 postinfection) of DT. Ten days post infection, shortly after the peak of viral replication, CD8^+^ T cell responses and viral loads in the spleen were analyzed. In all panels, FV-infected DEREG mice receiving DT (+DT, black bars) are compared with infected DEREG mice in which Tregs were not ablated (white bars). Statistically significant differences were calculated by the non-parametric *t* test and *p*-values are given in the figures. Six mice per group were analyzed in two independent experiments. (A) Absolute numbers of activated CD8^+^ T cells expressing the effector cell marker CD43 (over 90% of these cells were also positive for CD44 and negative for CD62L, confirming their effector phenotype [unpublished data]). (B) Absolute numbers of activated CD8^+^ T cells specific for an epitope in the FVgag gene (tetramer^+^). (C and D) Absolute numbers of CD8^+^ T cells expressing granzyme A or B, respectively. (E) Absolute numbers of CD8^+^ T cells expressing CD107a. (F) Spleen viral loads determined by an infectious center assay [4].

## Supporting Information

Figure S1 Depletion of Tregs in FV-infected mice expressing a DT receptor/GFP cassette under the control of the Foxp3 promoter by injection of DT. DEREG mice [9] that express the DT receptor under the control of the Foxp3 promoter were infected with FV, and Tregs were depleted starting at the time point of infection by five injections (days 0, 2, 4, 6, and 8 postinfection) of DT. Ten days postinfection, depletion of Tregs and other lymphocyte populations in the spleen were analyzed by flow cytometry. In all panels, FV-infected DEREG mice that received DT (+DT, black bars) are compared with infected DEREG mice in which Tregs were not ablated (white bars). (A) Shows a representative staining for Foxp3 and GFP in gated CD4^+^ T cells. Numbers in the upper quadrants represent the percentage of positive cells. (B) Absolute numbers of T cells (CD4^+^ and CD8^+^) and CD19^+^ B cells after Treg depletion. All experiments were performed with a group of four mice.
(1.05 MB TIF)

Figure S2 Cytokine responses of CD8^+^ T cells in mice infected with FV and experimentally depleted of Tregs. DEREG mice [9] were infected with FV and Tregs were depleted starting at the time point of infection by five injections (days 0, 2, 4, 6, and 8 postinfection) of DT. Ten days postinfection, cytokine responses of CD8^+^ T cell were analyzed by intracellular cytokine staining for IFNγ, TNFα, and IL-2 [15]. In the figure, naïve (non-infected), FV-infected non-depleted, and FV-infected DEREG mice receiving DT (+DT) were compared. Four mice per group were analyzed. Representative results for each group are shown. Numbers in the right section represent the percentages of positive cells.
(1.49 MB TIF)
