# Supplementary figures and images for: Regulatory T Cells Suppress Antiviral Immune Responses and Increase Viral Loads during Acute Infection with a Lymphotropic Retrovirus
Source: PLoS Pathog. 2009 Aug 28;5(8):e1000406. doi: 10.1371/journal.ppat.1000406 (PMC2727466; doi:10.1371/journal.ppat.1000406)

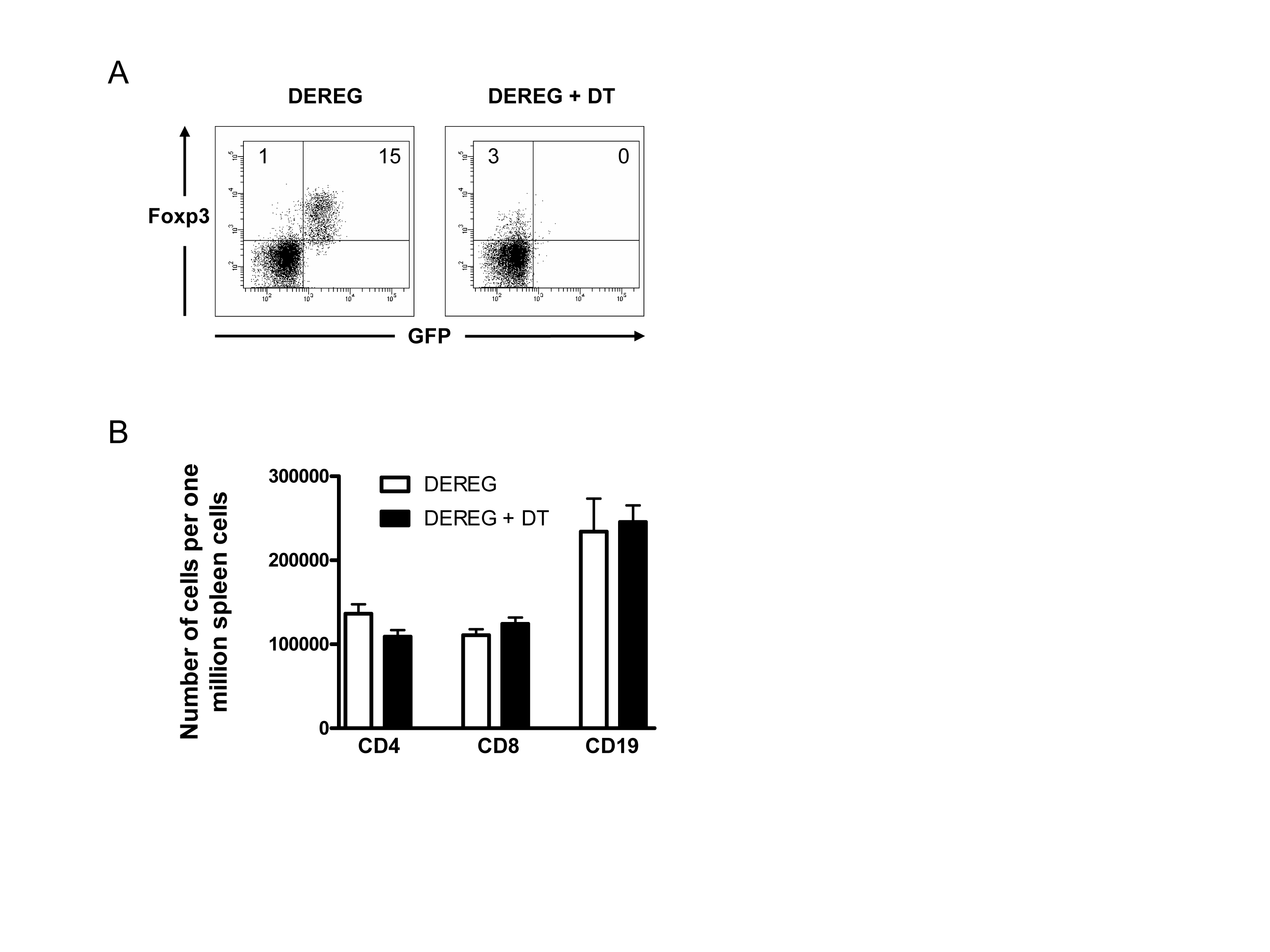

Supplement: Figure S1 — Depletion of Tregs in FV-infected mice expressing a DT receptor/GFP cassette under the control of the Foxp3 promoter by injection of DT. DEREG mice [9] that express the DT receptor under the control of the Foxp3 promoter were infected with FV, and Tregs were depleted starting at the time point of infection by five injections (days 0, 2, 4, 6, and 8 postinfection) of DT. Ten days postinfection, depletion of Tregs and other lymphocyte populations in the spleen were analyzed by flow cytometry. In all panels, FV-infected DEREG mice that received DT (+DT, black bars) are compared with infected DEREG mice in which Tregs were not ablated (white bars). (A) Shows a representative staining for Foxp3 and GFP in gated CD4+ T cells. Numbers in the upper quadrants represent the percentage of positive cells. (B) Absolute numbers of T cells (CD4+ and CD8+) and CD19+ B cells after Treg depletion. All experiments were performed with a group of four mice. (1.05 MB TIF) [file ppat.1000406.s001.tif]

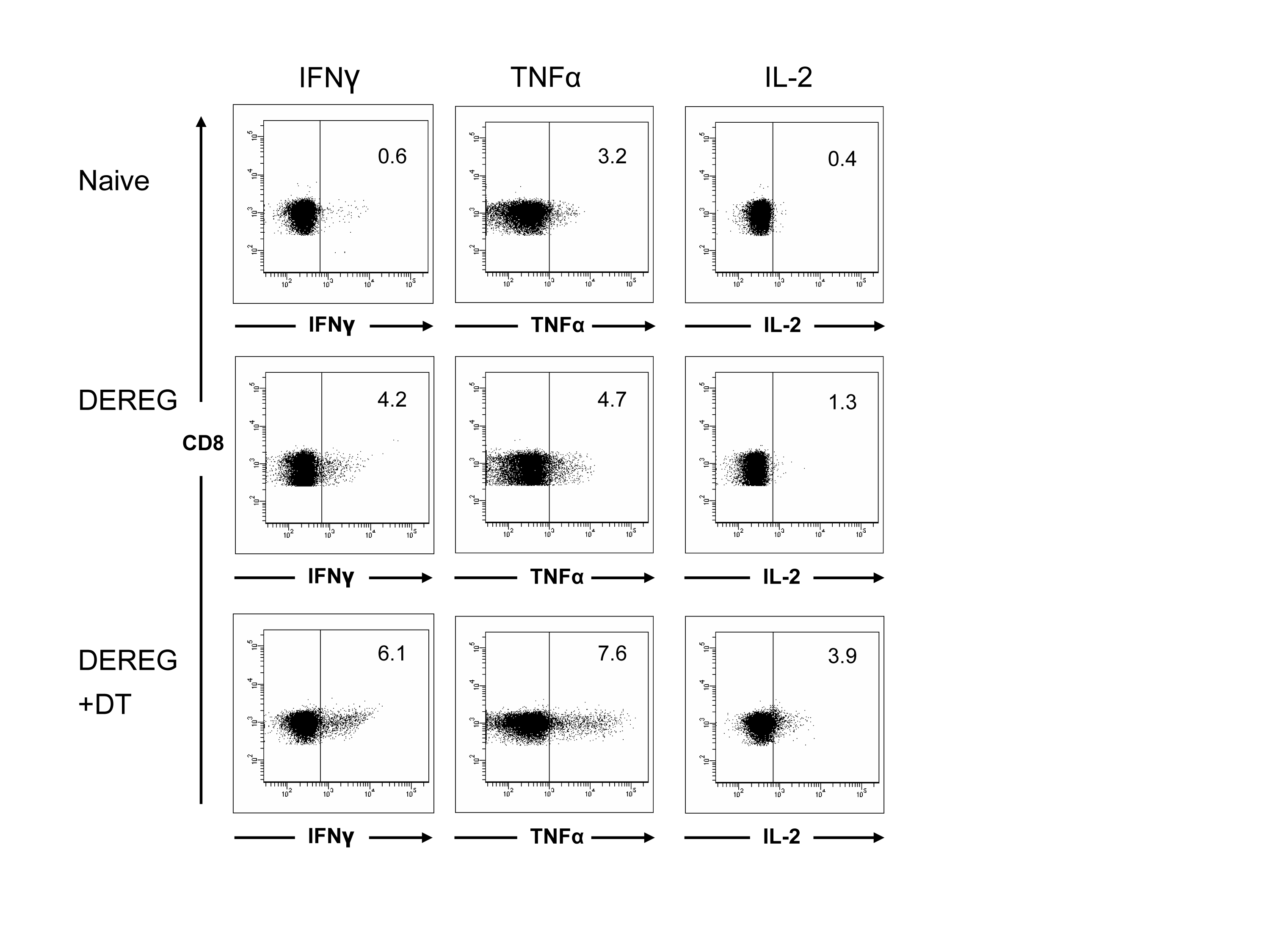

Supplement: Figure S2 — Cytokine responses of CD8+ T cells in mice infected with FV and experimentally depleted of Tregs. DEREG mice [9] were infected with FV and Tregs were depleted starting at the time point of infection by five injections (days 0, 2, 4, 6, and 8 postinfection) of DT. Ten days postinfection, cytokine responses of CD8+ T cell were analyzed by intracellular cytokine staining for IFNγ, TNFα, and IL-2 [15]. In the figure, naïve (non-infected), FV-infected non-depleted, and FV-infected DEREG mice receiving DT (+DT) were compared. Four mice per group were analyzed. Representative results for each group are shown. Numbers in the right section represent the percentages of positive cells. (1.49 MB TIF) [file ppat.1000406.s002.tif]
